# Foxp3^+^ Regulatory T Cells Restrain Th1 Response Shielding the Brain from Lethal Inflammatory Damage during Cryptococcal Meningoencephalitis

**DOI:** 10.21203/rs.3.rs-7142999/v1

**Published:** 2025-07-30

**Authors:** Hailong Li, Rylan Hissong, Kristie D. Goughenour, Yekateryna Sinitsyna, Maia Lintner, Brian Song, Heineken Q. Daguplo, Clifford S Cho, Anutosh Ganguly, Grace Y. Chen, Jessica C. Hargarten, Peter R. Williamson, Jintao Xu, Michal A. Olszewski

**Affiliations:** 1 Division of Pulmonary and Critical Care Medicine, Department of Internal Medicine, University of Michigan Health System, Ann Arbor, Michigan, USA; 2 Research Service, Ann Arbor VA Health System, Department of Veterans Affairs Health System, Ann Arbor, Michigan, USA; 3 State Key Laboratory for Diagnosis and Treatment of Infectious Diseases, NHC Key Laboratory of AIDS Prevention and Treatment, National Clinical Research Center for Laboratory Medicine, The First Hospital of China Medical University, China Medical University, Shenyang, 110001, China; 4 Division of Hematology and Oncology, Department of Internal Medicine, University of Michigan Health System, Ann Arbor, Michigan, USA; 5 Laboratory of Clinical Immunology and Microbiology, National Institute of Allergy and Infectious Diseases (NIAID), National Institutes of Health (NIH), Bethesda, Maryland, USA; 6 Center for Immunity and Inflammation, Rutgers New Jersey Medical School, Newark, New Jersey, USA; 7 Department of Pathology, Immunology and Laboratory Medicine, Rutgers New Jersey Medical School, Newark, New Jersey, USA

## Abstract

Inflammatory brain damage is an important factor contributing to mortality or lasting neurological sequelae in CNS infections, such as cryptococcal meningoencephalitis (CM), but little is known about natural immunoregulatory mechanisms in the infected brain. Here we report that regulatory T cells (Tregs) are a central immunoregulatory component in CM. Tregs are present within the CNS in both human CM patients and in the experimental murine CM. Treg-depletion exacerbates Th1-driven brain inflammation and neurological symptoms, accelerating mortality, despite enhanced fungal clearance in mouse CM. Aligned brain NanoString, scRNA-seq, and flow cytometry analyses revealed that Tregs reduce brain inflammation, especially T-cell recruitment activation and differentiation, shielding the brain from neurological damage. The major CNS-Treg recruitment appears to be chemokine receptor CCR8-mediated, supporting the importance of the CCR8/CCL1 axis in Treg recruitment into the brain. Tregs in CM are major producers of anti-inflammatory IL-10 and the growth factor Amphiregulin (Areg), which is implicated in neuronal repair. IL-10 deletion in murine CM phenocopies Treg depletion. Areg deletion showed no survival effect; however, IFN-γ production by effector T cells in the brain was reduced, supporting Aregs as a potential internal regulator of Treg immunosuppressive function. Finally, a Treg-enhancing immunotherapy using low dose IL-2-immune complex treatment substantially improves mouse survival and neurological outcomes. Together, we identified that Tregs are crucial for neuronal protection in CM, predominantly via IL-10 production, the CCR8-CCL1 axis is an important CNS-Treg recruitment mechanism and Treg enhancement is a potential therapeutic strategy to mitigate immunopathology during CM, and possibly other forms of meningitis.

## Introduction

*Cryptococcus neoformans* (*C. neoformans*) continues to be the major global cause of central nervous system (CNS) infections, causing a life-threatening cryptococcal meningoencephalitis (CM)^1–3^ accounting for nearly 181,000 deaths per year^4–6^. While it is more prevalent in immunocompromised patients^7–11^, increased incidence of CM in previously healthy individuals is reported^12^. CM neurological symptoms and pathology are often linked to inflammation^2, 13^, which either result from immune reconstitution or a maladaptive immune response. Accordingly, these syndromes are classified as immune reconstitution inflammatory syndrome (c-IRIS) or post infection inflammatory response syndrome (c-PIIRS), both resulting in poor prognosis and complicated treatment^3, 8, 14–20^. Both syndromes are characterized by strong T-cell/Th1 components^18, 21, 22^ and represent an extreme case of collateral immune damage seen to some degree in most CNS infections. Thus, separate from microbiological control with antimicrobial drugs, appropriate handling of CNS inflammation becomes an important priority in treatment of CM and other CNS infections, with new targeted therapies shielding the brain from inflammatory injury needed.

T-cell immunity, including Th-lineage polarization and resultant Th-cytokine production is critical in cryptococcal pneumonia, with Th1/Th17 being required for protection and Th2 as detrimental^23–26^. However, not all paradigms regarding immune mediated-protection and immunopathology demonstrated during lung infection directly translate to infection in the brain^27, 28^. In patients with c-IRIS/c-PIIRS, elevated T-cell responses, including IFN-γ in the cerebrospinal fluid often correlates with a poor prognosis and brain damage^16, 29, 30^. In our CM/PIIRS mouse model, we found excessive Th1 inflammation and CXCR3^+^ T cells leading to brain damage and neurological pathology^21–24, 26, 31, 21^. In this scenario, CD4^+^ T cell depletion leads to improved symptoms and mouse survival, despite fungal expansion in the brain^23^. This uncovers a clinical conundrum where Th1/IFN-γ are crucial for fungal clearance, but also profoundly damaging to the brain. As such, a better understanding of the Th1 response in CM and novel therapies which target detrimental Th1-responses are needed.

Foxp3^+^ regulatory T cells (Tregs) are an anti-inflammatory T-cell subset, tempering intensity of the immune response and orchestrating resolution. The balance between Treg (Foxp3^+^) and conventional effector T cells (Teff) is important for preventing CNS pathology and neuroinflammation^32–35^, especially in autoimmune diseases such as multiple sclerosis (MS)^35, 36^ and its model--experimental autoimmune encephalomyelitis (EAE)^37, 38^. Aside from suppression of inflammatory responses, Treg cells can limit damage by promoting tissue repair^39, 40^. In CNS models of stroke, CNS Tregs have been also reported to suppress astrogliosis, and promote neuronal recovery and regeneration through the production of amphiregulin (Areg), the ligand for the epidermal growth factor receptor (EGF-R)^40, 41^. However, the knowledge about the role of Tregs in CNS infections and CM is very limited. In our previous studies of CM, frequencies of Tregs were increased in both CXCR3^−/−^ and CCR2^−/−^ mice compared to WT mice along with improved mouse survival^24, 26, 31^, hinting that that Tregs could be protective. Furthermore, Treg dysfunction was noted in cases of cryptococcal or non-cryptococcal IRIS, motivating our studies in this area. Here, we report that Tregs are present in the CSF from CM patients and accumulate in the brain of mice with experimental CM. Treg depletion enhances Th1-driven brain inflammation, worsens neurological symptoms and molecular readouts of brain injury, and accelerates mortality, despite a reduction in fungal burden. We find that Tregs are the major CNS source of IL-10, which is a major driver of protective anti-inflammatory effects in CM. Finally, we report that Treg-enhancing immunotherapy using a low-dose IL-2 complex therapy demonstrates unprecedented therapeutic effects, limiting inflammatory brain injury and substantially improving survival of mice with CM.

## Results

### Tregs are a crucial factor promoting survival of CM despite reduced fungal clearance.

**Extended data figure 1.**
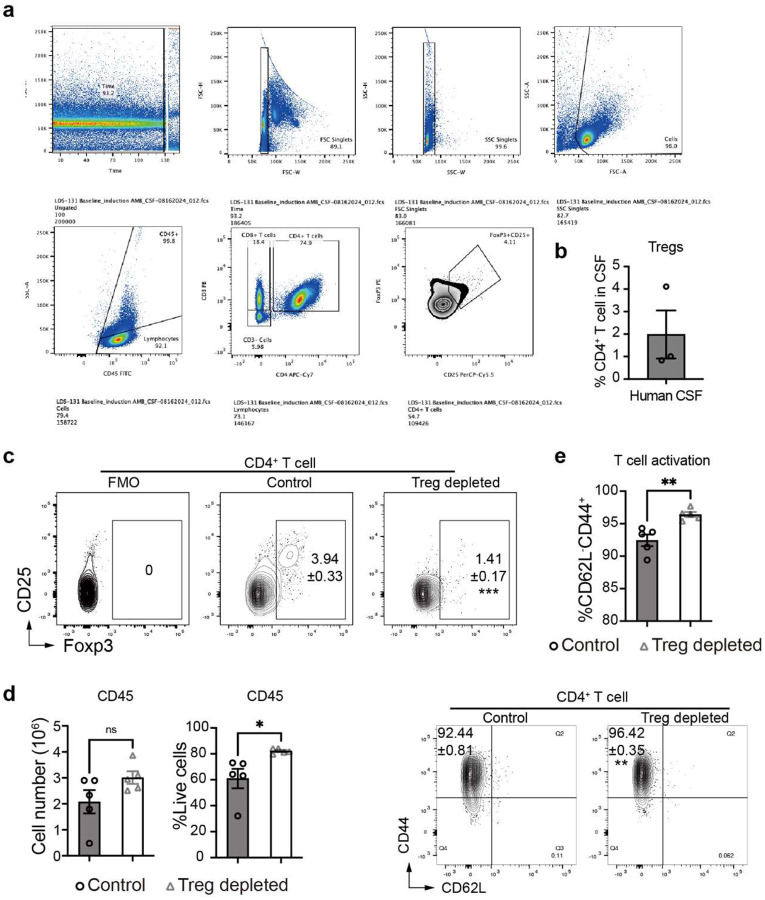
Gating strategy for the flow cytometry for CM patients. Gating strategy (**a**) and percentages (**b**) of Treg in the CSF from human patients. Treg suppression in CM mice treated using anti-CD25Ab led to a lower Tregs population in the brain (**c**), more CD45^+^ immune cell infiltration (**d**) and a higher CD4^+^T cell activation (CD44^+^CD62L^−^) (**e**), compared to WT mice. Results represent means ± SEM. *p < 0.05; **p < 0.01.

**Figure 1.**
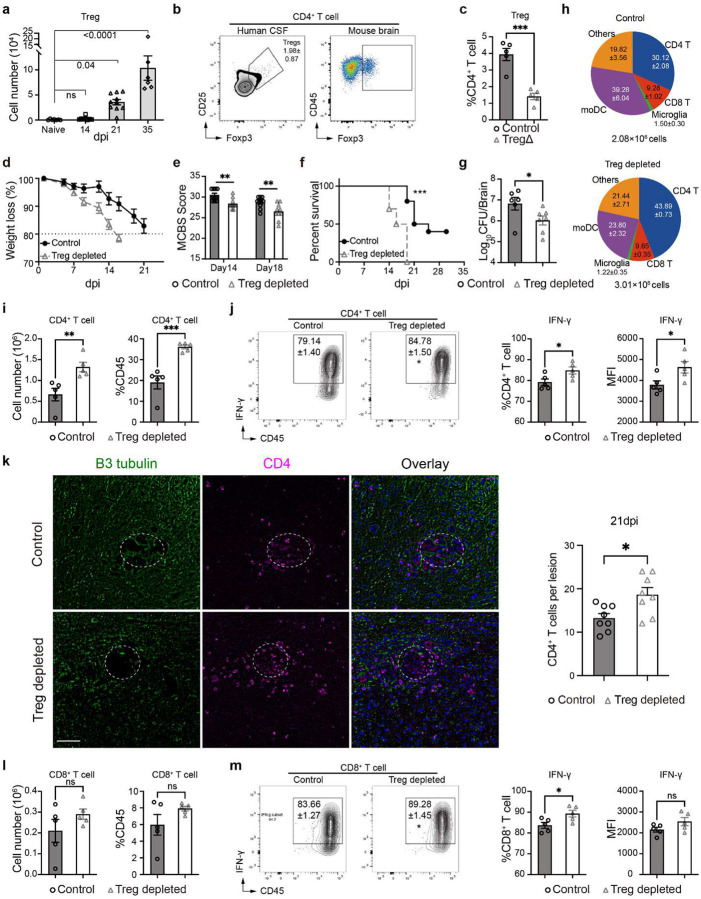
Foxp3^+^ regulatory T cells (Tregs) serve as essential shields of the brain from an excessive T-cell driven inflammation during *Cryptococcus* meningoencephalitis (CM). Mice, infected with *C. neoformans* 52D intravenously (10^6^ CFU) to induce CM were followed up to 35 days post infection (dpi): (**a**) Foxp3^+^ T cells detected in brain leukocyte isolate by flow cytometry at 0 (uninfected), 14, 21, and 35 dpi, showing time-dependent accumulation Tregs; (**b**) Tregs in both CSFs of human CM patients (Mean±SEM) and brains from CM mice; (**c**) Treatment of CM mice using anti-CD25Ab substantially reduced Treg frequencies compared to those treated with the isotype control. Treg suppression in CM mice with anti-CD25Ab resulted in: (**d**) accelerated weight loss, and (**e**) enhanced neurological symptoms assessed by reduced MCBS scoring at 14 and 18 dpi; (**f**) accelerated mouse mortality increasing to 100%; despite (**g**) accelerated fungal clearance shown for 21 dpi. Treg depletion altered cellular composition, especially an expansion of CD4^+^ T-cell compartment within the brain (**h**). T cell responses intensified through both increased total cell number (**i**) and further increase in IFN-γ production despite already highly Th1 polarized response in control mice (**j**). Immunofluorescent (IF)-microcopy with stained with β-III tubulin antibody to visualize neurons (green) and CD4^+^ T cells antibodies (red) shows increased accumulation of CD4^+^ cells around the lesions post Treg depletion (**k**). The effects on CD8 subset (**l**), while follows similar trends are less pronounced but showing significant increase in frequency of IFN-γ producing CD8^+^ cells (**m**). Quantitative data are shown individually and as mean±SEM (n=5–10 mice/group/time point). ANOVA with multiple comparisons, Student’s t tests, or log-rank test, were used as appropriate for data analysis (*p < 0.05; **p < 0.01; ***p < 0.005).

Immunological damage contributes significantly to morbidity and mortality in CNS infections. Tregs are an important factor opposing excessive inflammation and are capable of limiting Th2-driven lung pathology associated with pulmonary cryptococcosis^42^. Little is known about Tregs in CM, especially in the context of T-cell-driven immune damage as in patients with CM inflammatory syndromes (IRIS and PIIRS)^7, 18^. We studied the role of Tregs using our established CM mouse model, which is characterized by strong immune responses that lead to brain injury^23, 24^ and recapitulates that observed with human PIIRS and cIRIS^7, 18^. Foxp3^+^ Tregs accumulate in mouse brains during CM in a time-dependent manner that continuously increases until 35 days post-infection (dpi) (Fig. 1a). The Treg accumulation is delayed relative to Teff recruitment^23^, but the Treg recruitment wave marks the end of mortality in the CM mice and beginning of resolution^23^. The presence of Tregs was also validated in the CSF of a CM patient with PIIRS (Figs. 1b, Extended data fig. 1a, b), supporting the notion that Treg/Teff interplay is important for the balance of pro- and anti-inflammatory responses in patients with CM. To establish Treg functionality in CM, Treg depletion was carried out using anti-CD25 antibodies in CM mice, which substantially reduced the percentage of Tregs in the brain at 21 dpi (Figs. 1c, Extended data fig. 1c). Following this, Treg depletion led to worsening of neurological symptoms as determined by accelerated bodyweight loss (Fig. 1d), lower murine coma and behavior score (MCBS) (Fig. 1e), and more rapid mouse mortality (Fig. 1f), despite enhanced fungal clearance (Fig. 1g). These data thus support the central role of Tregs, presumably by protecting the host from inflammatory brain damage during CM.

### Tregs shield the brain from excessive Th1 inflammation and severe brain damage during CM.

**Extended data figure 2.**
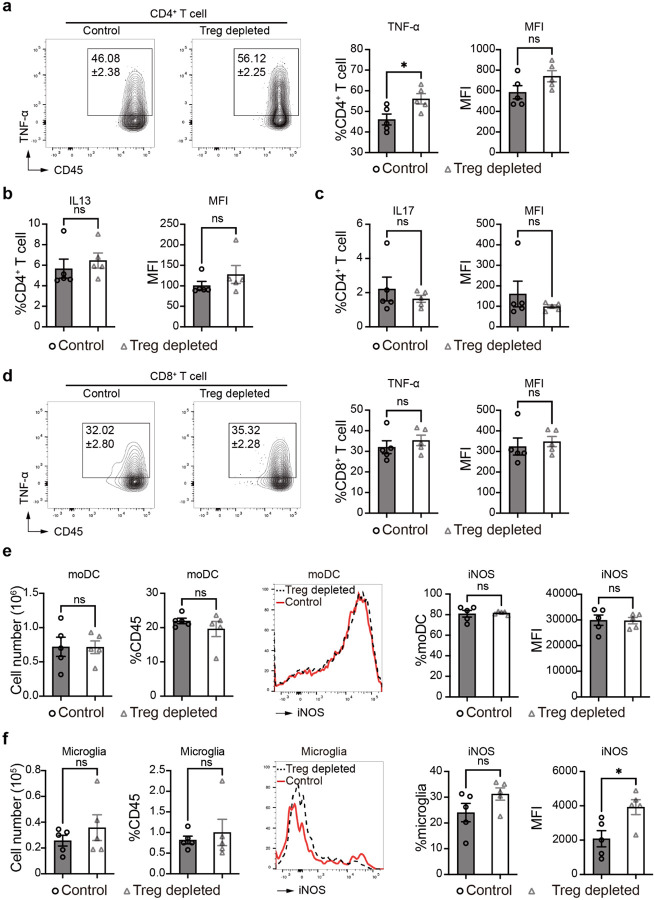
Effects of Treg depletion on main leukocyte subsets activation in the CM brain. Leukocytes from infected mice were isolated and analyzed by flow cytometry. Cytokines associated with Th1, Th2 and Th17 lineages were evaluated at 21dpi in CD4^+^ T cells. TNF-α (Th1) production by CD4^+^ T cells was elevated in Treg depleted mice (**a**), but no effect on IL-13 (Th2) or IL-17 (Th17) was observed (**b-c**). TNF-α production by CD8^+^ T cells was also not altered by Treg depletion (**d**). There was no effect on moDCs recruitment or their iNOS production, or the numbers of microglia but microglia iNOS expression was increased in Treg depleted mice (**e-f**). Data are shown individually and as mean±SEM (n=5 mice/group/time point). Student’s t tests were used (*p < 0.05).

To dissect the immunological basis of the protective functions of Tregs in CM brains we analyzed brain leukocytes from Treg-depleted and control CM mice. Treg depletion resulted in a greater immune CD45^+^ immune cell infiltration into brain (Extended data fig. 1d) especially CD4^+^ T-cells, including substantial expansion of their relative (Fig. 1h) and absolute (Fig. 1i) numbers in the brain. In addition, brain CD4^+^ T cells possessed an enhanced activation status (Extended data fig. 1e), increased IFN-γ and TNF-α production (Figs 1j, Extended data fig. 2a) following Treg depletion, supporting further strengthening of Th1 brain inflammation in the absence of Treg cells. Consistently, production of Th2 (Il-13) and Th17 (IL-17A) cytokines by Teff in mouse brains was limited and not altered by Treg depletion (Extended data fig. 2b–c). Histologically, the density of infiltrates with CD4^+^ cells were increased in Treg-depleted mice, at the perimeters of cryptococcal microcysts (Fig. 1k), where the greatest inflammatory damage to the brain is typically noted^31^. Treg-depletion affected the CD8^+^ T-cell subset to a lesser degree, although similar trends were observed (Figs. 1l–m, Extended data fig. 2d). We did not see an effect of Treg depletion on the number or frequency of MoDCs and their iNOS production at 21dpi (Extended data fig. 2e); however, Treg depletion increased iNOS production by microglia, without impacting the number of microglia in the brain during CM (Extended data fig. 2f). Thus, Tregs profoundly regulate CNS inflammation in CM; in particular, CD4^+^ T-cells and their Th1 activation, which in excess, is highly detrimental during CM^23, 24^.

### Treg depletion amplifies dysregulated gene expression, enhancing neuroinflammation and neurodegeneration markers in the CM brain.

**Extended data figure 3.**
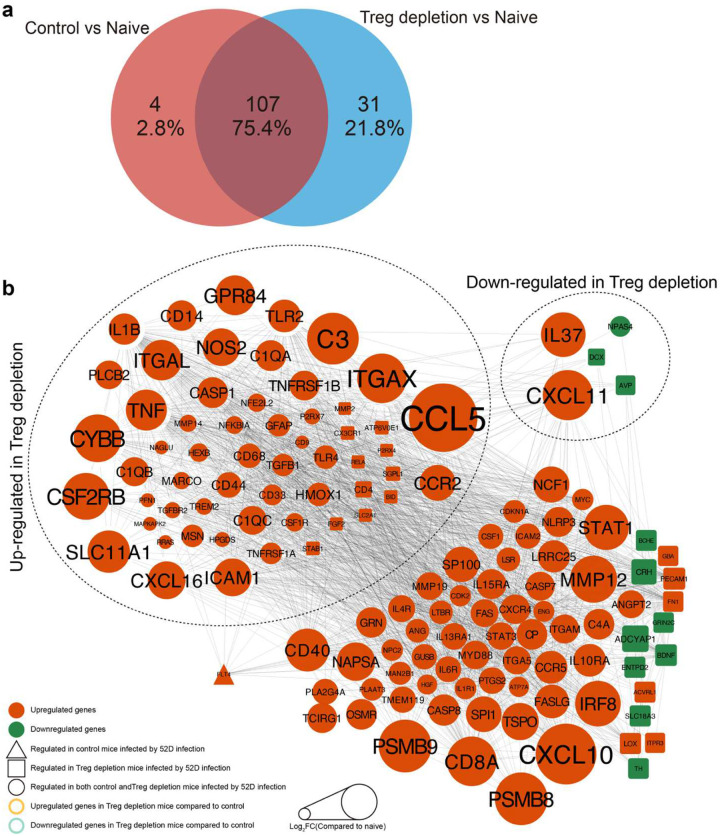
Analysis of Treg depletion effect on relevant gene transcription using neuroinflammation and neurodegeneration RNA panel (NanoStrings) in the CM brain. Differentially expressed genes in Naïve, Control-CM and Treg depleted-CM brain mRNA were analyzed (foldchange>2, p value<0.05) (**a**); and used for Protein-protein interactions (PPI) analysis. (**b**).

**Figure 2:**
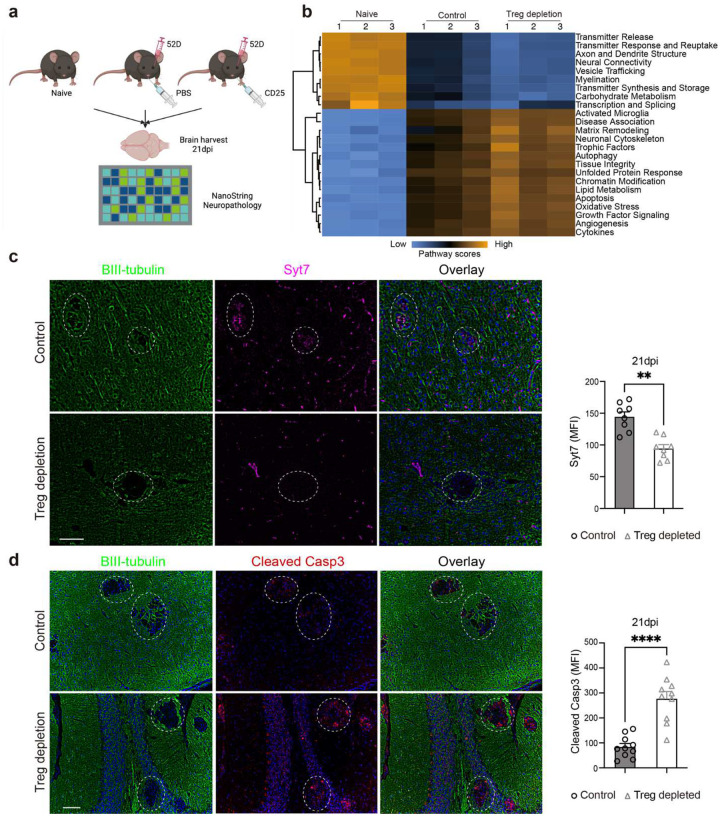
Treg suppression worsens pathological neuroinflammation and neuronal damage during CM. Brains from naïve (naïve), CM control (Control) and anti-CD25-treated (Treg-depletion) mice were harvested at 18 dpi for RNA extraction followed by NanoString analysis (Mouse Neuropathology Panel), and the differentially expressed genes were used for analysis (**a**); Note the amplification of pathological responses, i.e. greater suppression of neuronal connectivity pathways, and further increase of neurodegeneration and neuroinflammation pathways in mouse brains following Treg depletion (**b**). Immunofluorescent analysis of neuronal status (**c**) and apoptosis (**d**) in mouse brain at 21 dpi stained with antibody to neuronal β-III tubulin, green, synaptic protein, synaptagmin-7 (Syt7), purple, and apoptosis marker, red. Note enhanced depletion of Syt7 and increased cleaved caspase-3 detection in neurons neighboring cryptococcal lesions in anti-CD25-treated mice. N=3 mice per group for NanoStrings and minimum 2 pre group for histology (8–10 MFI assessment for each staining at different areas of brain section). **p < 0.01, ****p < 0.001.

To evaluate the impact of Tregs on brain functionality during CM, a NanoString neuropathology multiplex panel was employed to quantify the expression level of 770 genes related to CNS health and disease. Comparisons were made between naïve mice (Naïve) and CM mice treated with either isotype (Control) or anti-CD25 antibody (Treg depletion) (Fig. 2a). The suppression of pathways supporting neuronal connectivity and function, and upregulation of neurodegeneration and neuroinflammation pathways in CM mice (Fig. 2b) were further amplified in Treg-depleted mice, including aggravated neuronal stress responses and apoptosis (Fig. 2b). Compared to naïve mice, 111 and 138 differentially expressed genes (fold-change>2, p value<0.05, differential expressed genes (DEGs)) were found in Control vs Naïve and Treg-depleted vs Naïve, and 107 DEGs were shared (Extended data fig.3a, b, Extended data table). For example, CD4, CD8A, IL1R1, TNF-α, and NOS2 (inflammatory) and IL10, IL2RA (regulatory) genes were induced in the brain by *C. neoformans* infection; however, TNF-α, NOS2, CD4, CD14, IL1B were further upregulated following Treg depletion (Extended data fig. 3a, b, Extended data table). *In situ*, the neuronal connectivity protein, Synaptotagmin-7 (Syt7), reduction of which within the CM lesion perimeter correlates with the level of brain damage^21, 31^, was significantly decreased in Treg-depleted mice compared to control CM mice (Fig. 2c). Likewise, cleaved caspase-3, which marks areas of neuronal death in CM mice^24^ was increased in Treg depleted CM mice (Fig. 2d). Thus, Tregs were found to prevent inflammatory brain damage in CM by opposing degenerative neuroinflammatory processes in the *C. neoformans*-infected brain.

### The chemokine CCR8-CCL1 axis contributes to Treg recruitment into murine brain during CM.

**Extended data figure 4.**
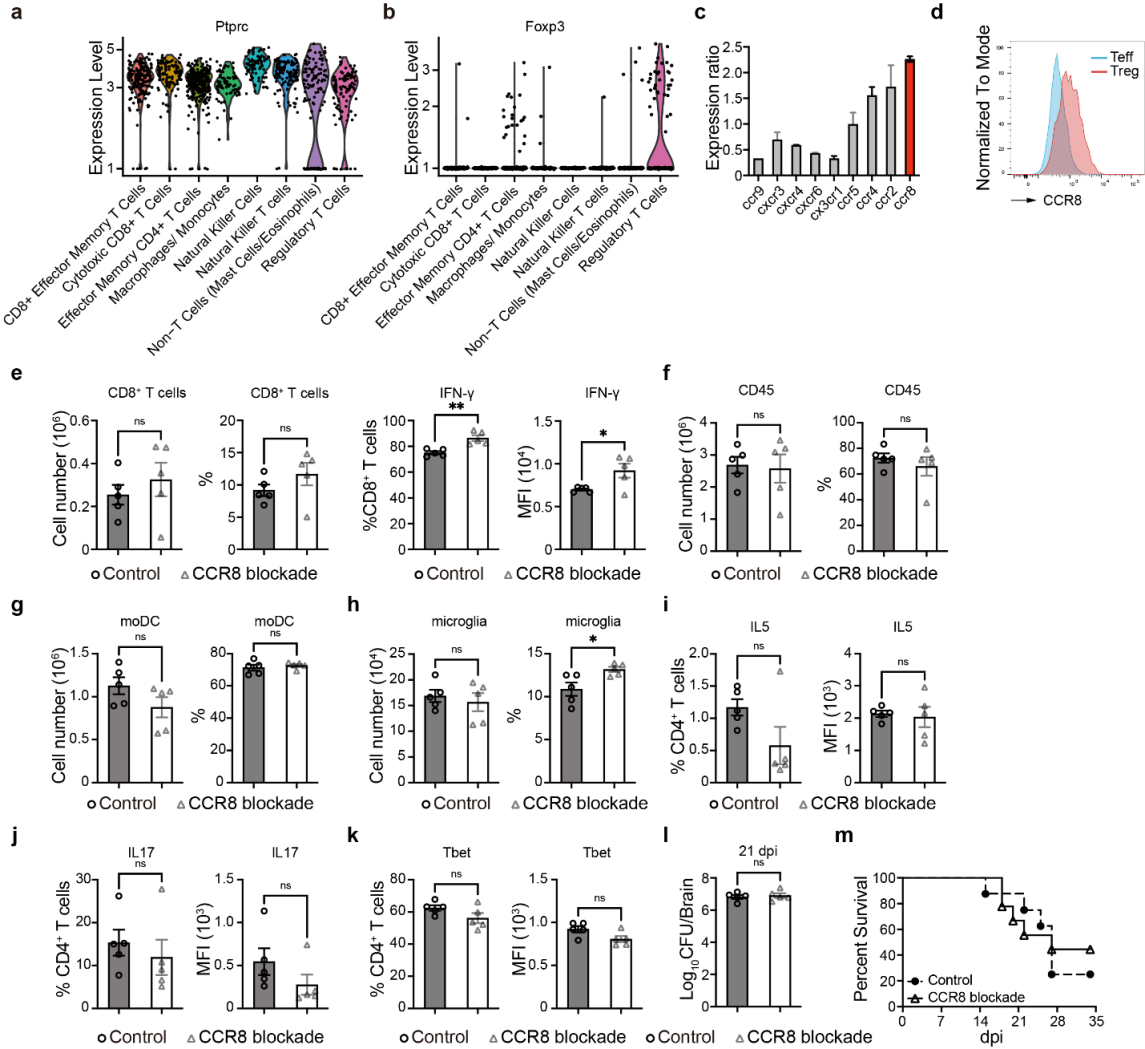
CCR8 expression and the effect of CCR8 blocking with CCR8Ab during CM. Violin plots of Ptprc (CD45) and Foxp3 of scRNAseq data (**a-b**) showing preferential expression of CCR8 mRNA in Tregs. Ratios of Tregs and T effector cells for major chemokine gene expression shows that Ccr8 is the most upregulated chemokine receptor in Tregs compared to Teff (**c**), and Flow cytometry shows much stronger surface expression of CCR8 on Tregs in the brain (**d**). CCR8 blocking resulted in higher IFN-γ production by CD8^+^ T cells (**e**), but had no impact on brain infiltrating CD45^+^ leukocytes (**f**), moDC (**g**) but increased microglia (**h**). No effect was found for studied CD4^+^ T-cell cytokine or Tbet expression (**i-k**), brain fungal burden (**l**) or mouse Survival (**m**) post- CCR8 blockade. Results represent means ± SEM (n=5–16). *p < 0.05; **p < 0.01.

**Figure 3:**
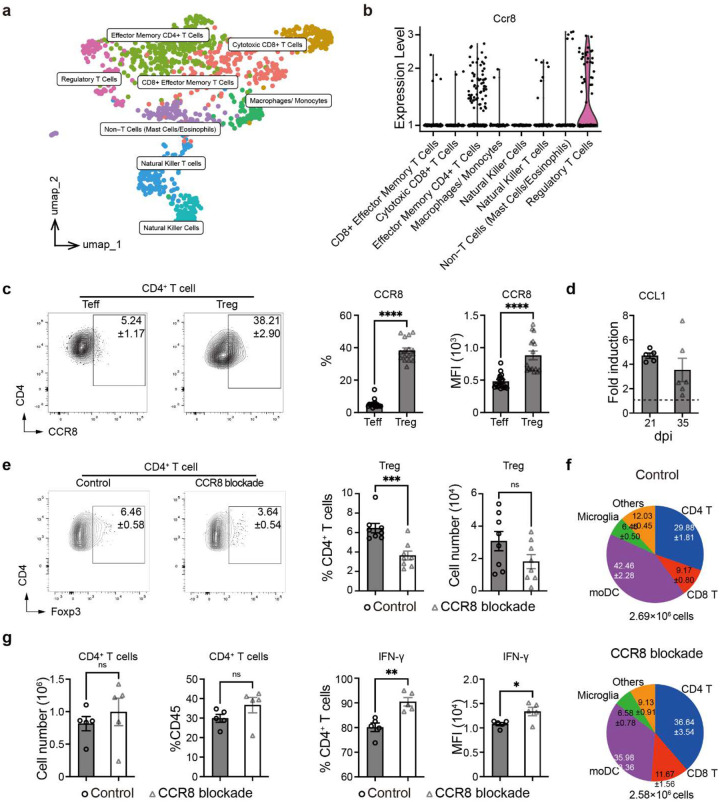
Chemokine CCR8-CCL1 axis contributes to Treg accumulation in the brain during CM. The single cell RNAseq analysis revealed (**a**) Diversity of T cell subpopulations with a distinct Treg cluster visualized by UMAP analysis, violin plots of CCR8 expression analysis in cell clusters shows preferential expression of CCR8 by Tregs (**b**). High CCR8 surface expression occurs on Treg but not Teffs within the CM brain (**c**) and concurrent upregulation of Ccl1 (a CCR8 ligand) at the timepoints of the Treg brain migration 21–35 dpi (**d**). The CCR8 antibody (Ab) treatment decreased frequencies of Tregs and resulted in a trend towards an expansion of CD4^+^ cell fraction in the CM brain (**e, f**), and increased IFN-γ production on CD4^+^ T cells (**g**). Results represent means ± SEM, (n=5–16). *p < 0.05; **p < 0.01; ***p < 0.005; ****p < 0.001.

To identify a unique gene set expressed by Tregs, the transcriptional profiles of Tregs and Teffs isolated from brain during CM were compared. Among T cell subpopulations and other inflammatory cells, we distinguished a Treg cluster apart from the conventional T-cell clusters in the CM brain. (Figs. 3a, Extended data fig. 4a–b; Extended data table). Among DEGs, we found that the chemokine receptor Ccr8 was preferentially expressed on Tregs (Fig. 3b). These findings were validated using RT-qPCR (Extended data fig. 4c) and by flow cytometry (Figs. 3c, Extended data fig. 4d). The major CCR8 ligand, Ccl1 was also ~4-fold upregulated in the brain at multiple timepoints of CM (Fig. 3d), suggesting the importance of the CCR8-pathway for Treg recruitment into the brain. We evaluated this using systemic anti-CCR8-blocking antibody treatment in CM mice, which resulted in suppressed recruitment of Tregs into the brain (Fig. 3e). Similarly, we also saw changes in the brain immune cell composition following similar trends observed in the Treg depletion study (Fig. 3f), specifically, a trend towards increased total CD4^+^ T-cell number and increased IFN-γ production in both in CD4^+^ T cells and CD8^+^ T cells (Fig. 3g, Extended data fig. 4e). No impact on total CD45^+^ cells, moDC, or microglia (Figs. Extended data fig. 4f–h) or changes in Th2 and Th17 CD4 T-cell differentiation (Extended data fig. 4i–k) were noted. Finally, brain CFU and mouse survival were not impacted by CCR8 blockade (Extended data fig. 4l–m). Thus, our data demonstrates that the CCR8-CCL1 axis plays a role in Treg recruitment into the brain during CM; however, this effect was partial, and Tregs remain protective in the brain even during inhibition of CCR8 signaling.

### Murine IL-10 gene deletion phenocopies major findings of Treg depletion during CM.

**Extended data figure 5.**
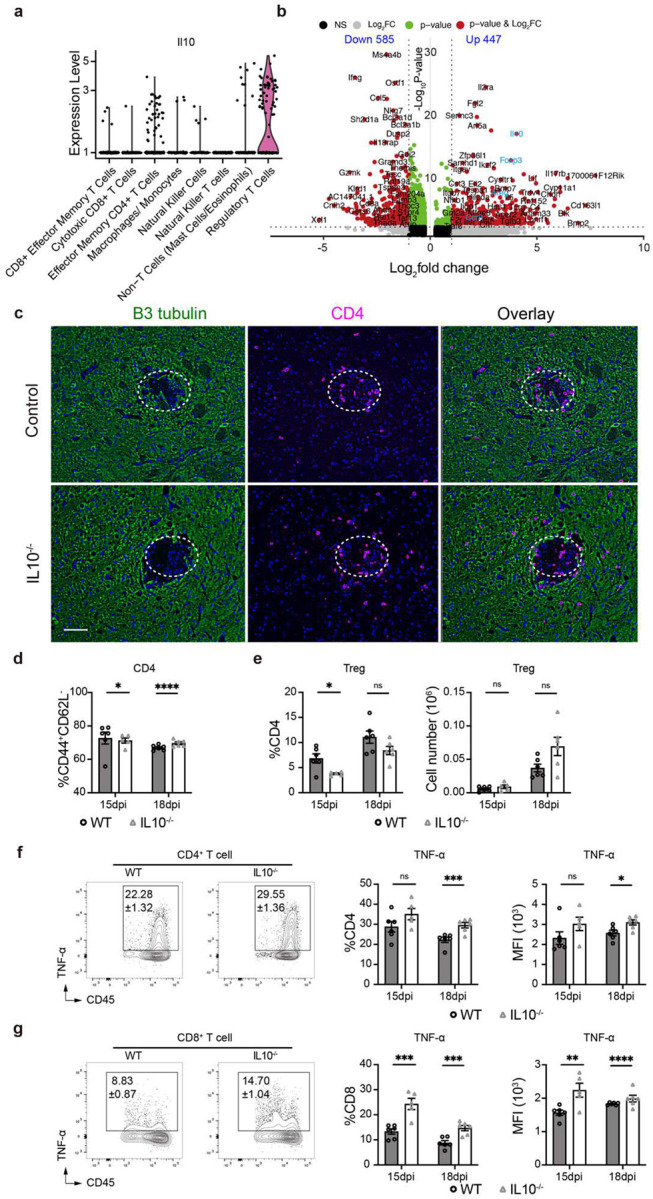
IL-10 expression in the brain and the effect of Il10 gene deletion on the course of CM in mice. Violin plot of Il10 gene expression from single cell profiling shows that Tregs are the major cell subset producing IL-10 in the brain (**a**). Volcano plot of gene expression between Tregs and Teffs using single cell profiling (**b**). Immunofluorescent with stained with β-III tubulin (green) and CD4^+^ T cells (red) shows increased accumulation of CD4^+^ cells around the lesions in IL10^−/−^ mouse (**c**). IL10^−/−^ showed enhanced CD4^+^ T cell activation (**d**), decreased Treg percentage at early CM stage, but no effect at late stage (**e**), increased TNF-α production in CD4 (**f**) and CD8 (**g**) T cells. Quantitative data are shown individually and as mean±SEM (n=5–6 mice/group/time point). (*p < 0.05; **p < 0.01; ***p < 0.005; ****p < 0.001).

**Extended data figure 6.**
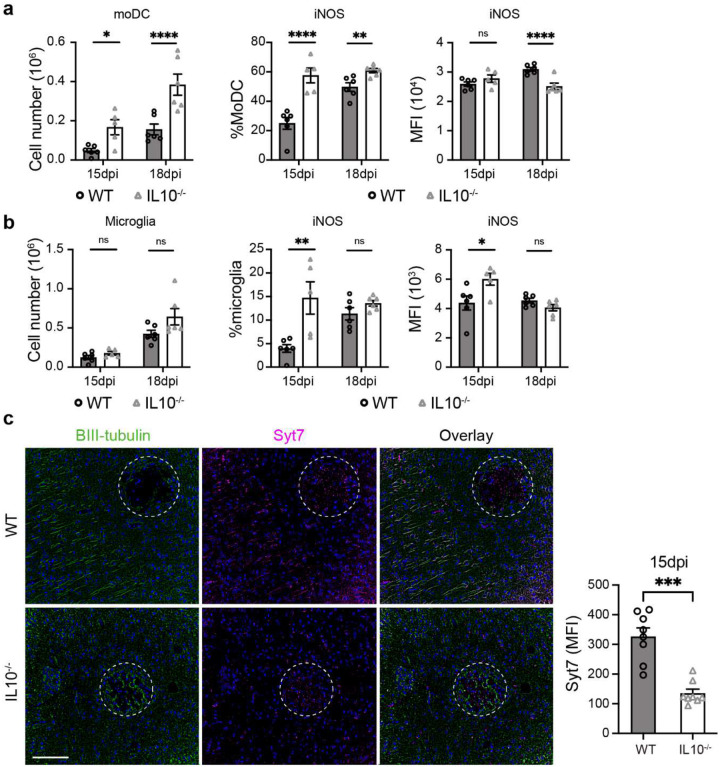
IL10 deletion leads to greater myeloid cell recruitment and more profound brain damage. Note significantly higher moDC numbers in the brain and their iNOS production in IL-10^−/−^ mice with CM (**a**), not impact on the microglia population size but their increased iNOS production (**b**). Immunofluorescent analysis shows accelerated neurological damage by accelerated depletion of Syt7 around the cryptococcal lesions IL10^−/−^ mice (15 dpi) (**c**). Quantitative data are shown individually and as mean±SEM (n=5–6 mice/group/time point). (*p < 0.05; **p < 0.01; ***p < 0.005; ****p < 0.001).

**Figure 4.**
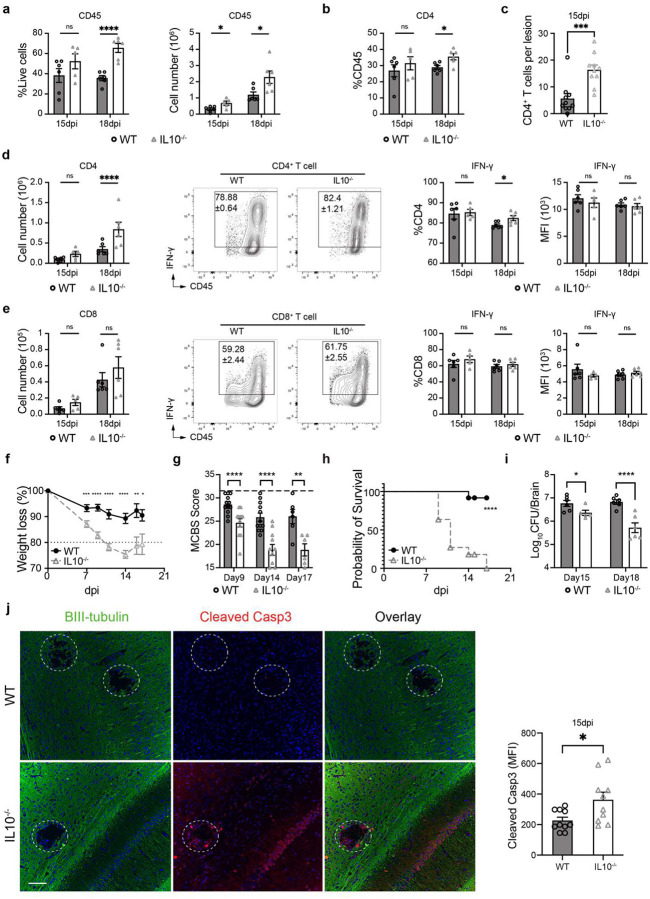
Murine IL10 gene deletion phenocopies Treg depletion during CM. WT C57BL6 and IL10^−/−^ mice were infected and analyzed as above. Murine IL-10 gene deletion resulted in more pronounced inflammatory response including: increased leukocyte infiltration into the brain (**a**); expansion of CD4^+^ T-cell subset and their enhanced IFN-γ response (**b, c, d**), but no significant change in CD8^+^ T cell and their IFN-γ^+^ subset (**e**); The IL-10^−/−^ mice with CM displayed accelerated decline of weight loss (**f**) and behavioral score (**g**), experiencing rapid, 100% mortality (**h**) despite accelerated fungal clearance (**i**). Immunofluorescent analysis showed accelerated apoptosis by accelerated the expression of cleaved caspase-3 around the cryptococcal lesions IL10^−/−^ mice (15 dpi) (**j**). Quantitative data are shown individually and as mean±SEM (n=5–10 mice/group/time point). *p < 0.05; **p < 0.01; ***p < 0.005; ****p < 0.001).

The IL-10 cytokine is a major anti-inflammatory factor produced by immune cells and is an effector for Treg function^43^. Our single cell transcriptome data showed that in the CNS, Tregs are the major source of IL-10 in the CNS during CM (Extended data fig. 5a–b, Extended data table), which motivated our studies to evaluate the importance of IL-10 in CM. Wildtype (WT), C57BL/6 and IL-10^−/−^ mouse were infected, analyzed, and compared as above. Similarly to Treg-depletion in CM mice (Fig. 1), IL-10 gene deletion promoted CD45^+^ leukocyte infiltration into the brain (Fig. 4a) and an expansion of the CD4^+^ T-cell component, increasing their proportion relative to that in WT-CM mice (Figs. 4b, c, Extended data fig. 5c). On day 18, CD4^+^ T cell numbers nearly doubled in IL-10^−/−^ mice compared to WT mice, and production of IFN-γ and TNF-α was increased, whereas there were no differences in Tregs (Fig. 4d, Extended data fig. 5d–f). Just as in Treg-depleted mice, we did not see an effect of IL-10 deletion on CD8^+^ T cell numbers, but interestingly, a more pronounced effect on TNF-α and no effect on IFN-γ production by CD8^+^ T cells was observed (Figs. 4e, Extended data fig. 5g). Consistent with accelerated Th1-inflammation in the brain, we also found increased recruitment of MoDC and iNOS upregulation on day 15 in these cells (Extended data fig. 6a). Microglia number was not affected by IL-10 deletion, but we observed accelerated upregulation of iNOS production in microglia (Extended data fig. 6b) as in Treg-depleted mice. Likewise, we observed an accelerated weight loss (Fig. 4f), progressively worsening neurological status (Fig 4g), and 100% early mortality in IL-10^−/−^ CM mice (Fig. 4h), despite increased fungal clearance (Fig. 4i). The IL-10^−/−^ mice CM pathology mirrored that of Treg-depleted mice, including intense staining of cleaved caspase-3 in neurons (Fig. 4j) and severe depletion of Syt7 at the perimeter of cryptococcal lesions (Extended data fig. 6c). Thus, murine IL-10 deletion closely phenocopied Treg depletion in CM, supporting a critical role of Treg-produced IL-10 in protecting the CNS from inflammatory damage during CM.

### Areg produced by Tregs in the brain counter-regulates anti-inflammatory Treg function without affecting CM symptoms.

**Extended data figure 7.**
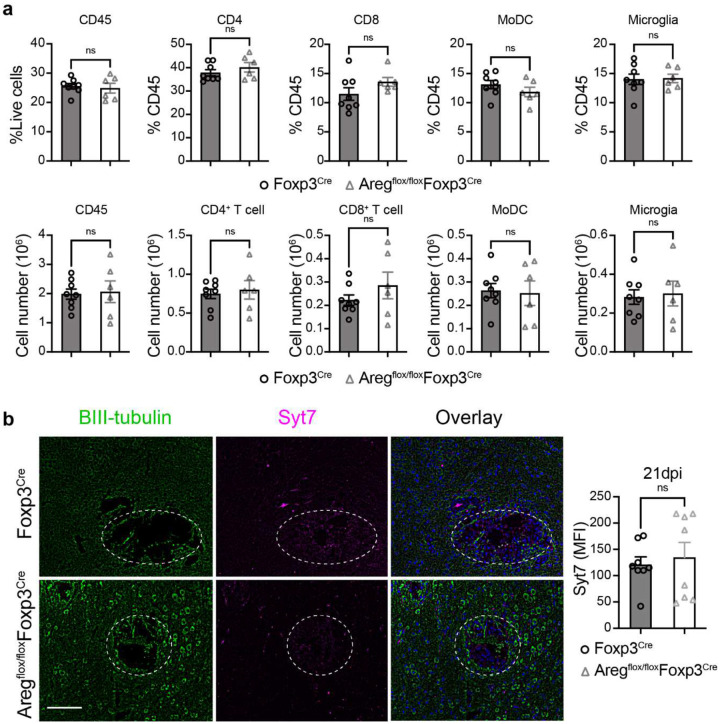
Areg expression by Treg does not affect leukocyte populations in the brain during CM or the level of neuronal damage. Areg deletion on Treg did not impact brain immune cell composition during CM, including percentage of CD45^+^ immune cells, CD4^+^ T cells, CD8^+^ T cells, moDC, and microglia at 35 dpi (**a**), and also had no impact on neurological damage evaluated using immunofluorescent analysis mouse brain stained with antibody to β-III tubulin neurons and synamptagomin-7 (Syt7) at 21 days postinfection (**b**). Results represent mean ± SEM (n=8 for each time point). ns p > 0.05.

**Figure 5.**
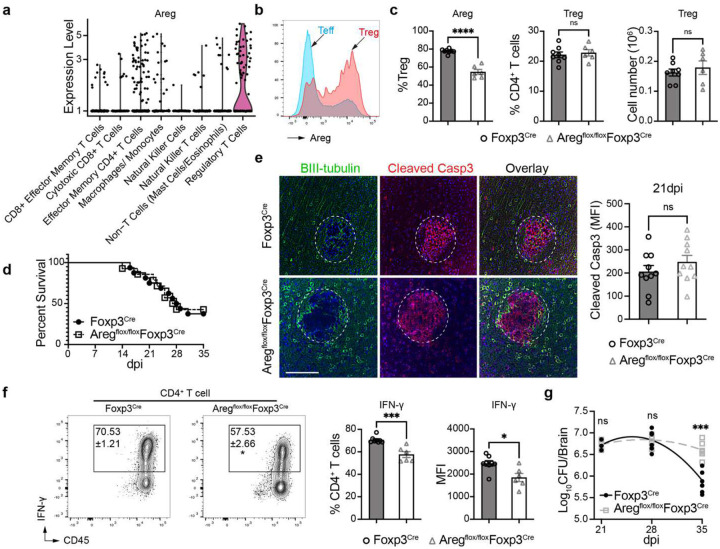
Amphiregulin (Areg) produced by Tregs counter regulates Treg function without CM affecting symptoms. The Areg in CM brain is preferentially expressed by Tregs relative to other leukocyte/T-cell subsets (**a-b**). Selective restriction of the Areg in Treg (tamoxifen-induced Areg^f/f^Fopx3^Cre-ERT2^ mouse) resulted in significant suppression of Treg-Areg expression without affecting Treg percentage and total numbers (**c**). Areg deletion did not affect mouse survival rate or the severity of cryptococcal lesions in CM (**d**), and Immunofluorescent analysis showed a comparable apoptosis with the Fopx3^Cre-ERT2^ control mouse (21 dpi) (**e**). but it suppressed IFN-γ production in CD4^+^ T cells (**f**) which slowed down the fungal clearance rate in the brain (**g**). Quantitative data are shown individually and as mean ± SEM (n≥6 mice/group). *p < 0.05; ***p < 0.005; ****p < 0.001.

While IL-10 produced by Tregs in the brain appears to be a major factor protecting the brain from inflammatory injury, we also found that amphiregulin (Areg) is highly expressed in Tregs (Figs. 5a–b). As Areg has been reported to play tissue repair and immunoregulatory roles in diseases such as lung fibrosis and cancers^32, 41^, we asked whether Areg produced by Tregs is also neuroprotective in CM. Using Tamoxifen-treated Areg^flox/flox^Foxp3^Cre-ERT2^ mice, we achieved substantial Areg-suppression in Tregs in the brain (Fig. 5c). As expected, this did not affect Treg or Teff population sizes (Extended data fig. 7a). Furthermore, Areg suppression did not change the magnitude of CD45^+^ immune cells recruitment or composition in the brain, specifically, CD4^+^ and CD8^+^ T cells, MoDC, or microglia (Extended data fig. 7a). Surprisingly, we did not observe any difference in survival (Fig. 5d) or brain pathology between Foxp3^Cre-ERT2^ and Areg^flox/flox^Foxp3^Cre-ERT2^ mice. Our experiments revealed equivalent Syt7 depletion in the perimeter of cryptococcal lesions (Extended data fig. 7b) and expression of cleaved caspase-3 (Fig. 5e) between matched control and Areg^flox/flox^Foxp3^Cre-ERT2^ mice. While this would argue against a neuroprotective effect of Treg-derived Areg in the CNS, we saw an unexpected decrease in IFN-γ production by CD4^+^ T cells (Fig. 5f) and corresponding increase in brain CFU (Fig. 5g) in Areg^flox/flox^Foxp3^Cre-ERT2^ mice at a late infection stage. These data suggest that Treg-produced Areg can serve as a counter-regulator of Treg function, at least in the context of IFN-γ production by T-cells in cryptococcal-infected brains, supporting a multifaceted role for Areg in the CNS.

### Treg-enhancing IL-2 immune complex treatment decreases brain inflammation and protects mice from pathology and mortality during CM.

**Extended data figure 8.**
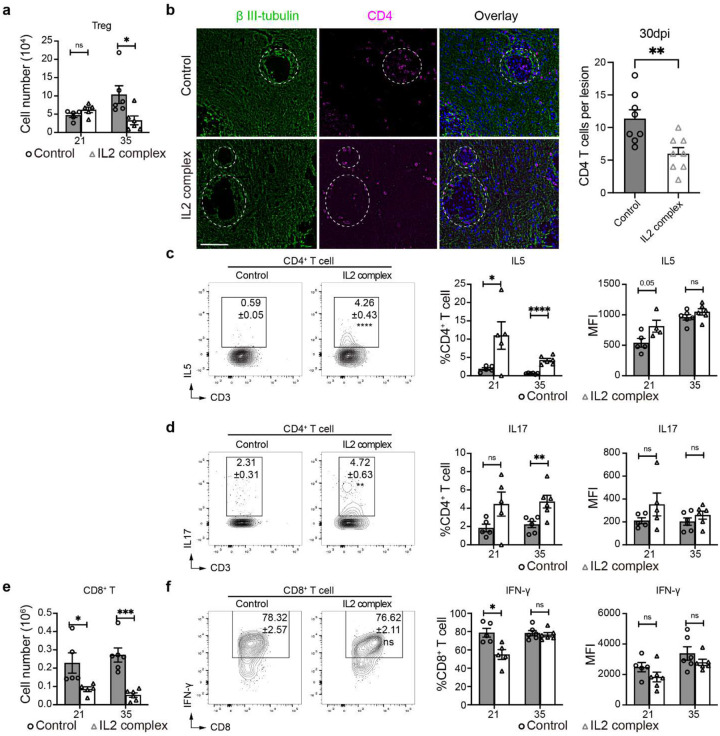
Treg enhancement therapy using IL-2 complex decreased Th1 polarization in the brain during CM. Treatment with IL-2 complex despite increasing Treg frequencies did not impact the total infiltrated Treg cell number (**a**) because of the profound suppression of total CD4^+^ T cell infiltration visualized here by IF microscopy (**b**). Furthermore, IL-2 complex treatment increased frequencies of of Th2 (IL-5) (**c**) and Th17 (IL-17) lineages of CD4^+^ T cells within brain infiltrate (**d**). CD8^+^ T cell recruitment and their IFN-γ production were decreased by IL-2 complex treatment (**e, f**). Quantitative data are shown individually and as mean±SEM (n=5–10 mice/group/time point). (*p < 0.05; **p < 0.01; ***p < 0.005; ****p < 0.001).

**Extended data figure 9.**
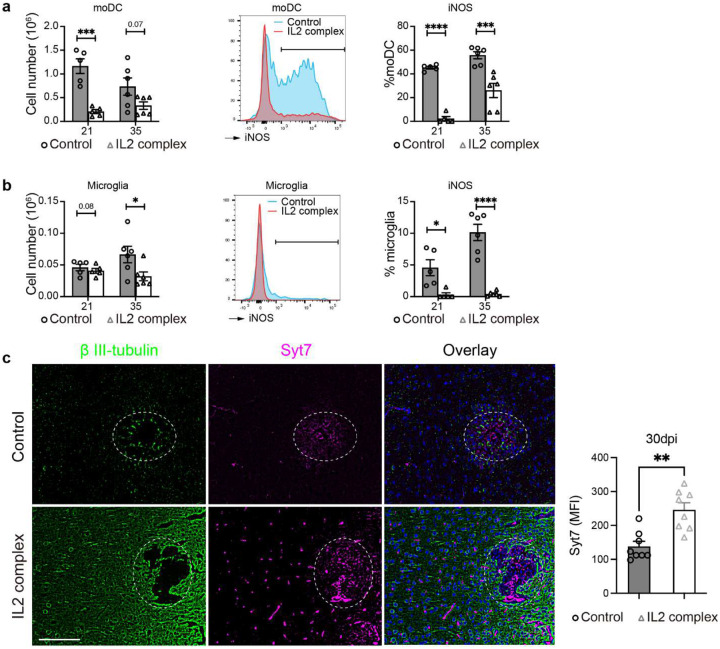
Treg enhancement therapy reduced mononuclear phagocyte numbers and activation and resulted in preserved synaptic protein Syt7 in the infected brain regions late in the infection. Treg enhancement using IL-2 complex therapy decreased recruitment of moDC and their iNOS production (**a**), and microglia and their iNOS production (**b**). Immunofluorescent analysis of mouse brain stained with β-III tubulin neurons and synamptagomin-7 (Syt7) showed a well-preserved Syt7 staining at the perimeter of the lesion of treated mice, even at 30 dpi in contrast with control mice showing significant Syt7 depletion at that time. Results represent mean ± SEM from one of three matched experiments (n=8–10 for each time point). ns p > 0.05, *p < 0.05; **p < 0.01; ***p < 0.005; ****p < 0.001.

**Figure 6.**
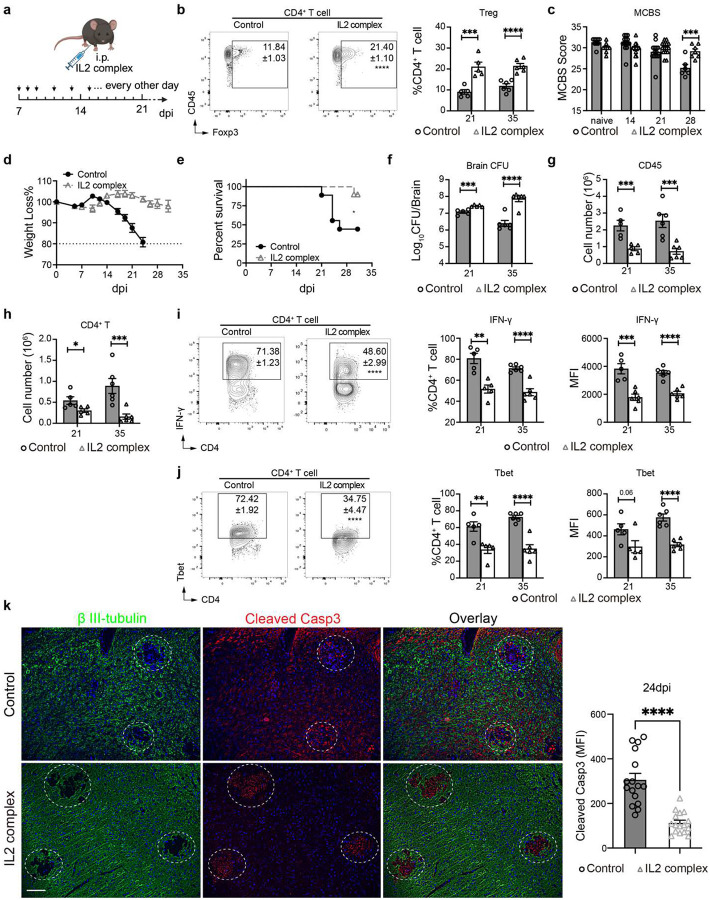
Treg expansion by IL-2-complex treatment reshapes brain immunology and protects mice from pathology in CM. The CM mice were treated with either isotype control or IL-2-Ab complex from 7 dpi once every other day (**a**). IL-2 complex treatment enhanced Treg frequencies in the brain during CM (**b**). This reduced neurological symptoms (**c**), weight loss (**d**), and drastically improved mice survival (**e**), despite opposing the fungal clearance (**f**). These effects were linked to a lasting suppression of CD45^+^ leukocyte migration to the brain especially (**g**), CD4^+^ T-cell recruitment (**h**) and their suppressed IFN-γ production (**i**) and Tbet expression (**j**). The Immunofluorescent analysis shows good preservation of apoptosis in proximity of cryptococcal foci, compared to untreated control. 24 dpi (**k**). N=5–10 mice/group/time point; *p < 0.05; **p < 0.01; ***p < 0.005; ****p < 0.001.

Having determined that Tregs play a protective role in a highly inflammatory CM, we explored the possibility of their therapeutic potential in CM. We employed an IL-2 immune complex treatment (Fig. 6a) to boost Treg function. This treatment in CM mice resulted in a profound and lasting enrichment of Treg proportions in the brain (Fig. 6b, Extended data fig. 8a) and resulted in a major improvement in animal condition, preventing a loss of behavioral scores and body weight and providing a substantial survival benefit (Figs. 6c–e), despite increasing brain fungal burden (Fig. 6f). This was mechanistically linked to a profoundly suppressed leukocyte recruitment, especially CD4^+^ T cells (Figs. 6g–h, Extended data fig. 8b). Apart from reduced numbers, CD4^+^ T cells shifted away from Th1-skewed (IFN-γ and Tbet) responses (Figs. 6i–j) with some enhancement of Th2 (IL-5^+^) and Th17 (IL-17^+^) subsets (Extended data fig. 8c–d). The recruitment of CD8^+^ T cells was also substantially blunted (Extended data fig. 8e), while their IFN-γ response was suppressed to a lesser degree at 21 and not at 35 dpi (Extended data fig. 8f). The effect of IL-2 therapy on MoDC was also profound. Their recruitment into the CNS was substantially blunted, and their iNOS expression was significantly delayed and reduced (Extended data fig. 9a). Likewise, microglia numbers were suppressed and their iNOS expression was completely ablated (Extended data fig. 9b). While this severe suppression of T cell and moDC came at the cost of a reduced fungal clearance (Fig. 6f), we found that IL-2-complex therapy protected brain integrity, marked by reduced neuronal caspase-3 (Fig. 6k) and much better preserved Synaptotagmin-7 (Syt7) at the perimeter of the fungal lesions within the CM brain, even at a very late timepoint (Extended data fig. 9c). Collectively, our data demonstrates that Tregs have therapeutic potential, protecting CNS from inflammation in CM, since their enhancement by IL2 complex treatment restricts inflammatory brain damage and improves CNS symptoms and mortality.

## Discussion

Excessive inflammation leads to severe brain damage in patients with CM, especially in those with PIIRS and IRIS, resulting in 30–70% mortality rates^1–3, 13, 20, 44^. Tregs are a population of immunoregulatory cells, tempering excessive inflammation responses^35, 36, 45–47^. Here, we investigated the impact of Tregs on the immune responses in the brain during CM, demonstrating that Tregs are critical players in the anti-fungal CNS response. By counter-regulating the Th1 immune response and controlling inflammation, Tregs shield neurons from lethal damage inflicted by the host’s own defenses during CM. We further defined that Treg recruitment to the brain relies in large part on the CCR8-CCL1 axis and demonstrated that two Treg factors, IL-10 and Areg, influence immune responses in the brain. Finally, we demonstrated the immunotherapeutic potential of Treg expansion by an IL-2 immune complex therapy in CM, which drastically improved neurological symptoms and survival in mice. Such immunotherapy could be beneficial in cPIIRS and cIRIS patients as an adjunct to antifungal therapy in CM and possibly other CNS infections.

Tregs recruited into the brain inhibit excessive Th1 immune responses and inflammatory damage. In our CM model, we observed an accumulation of Tregs in mouse brains in a time-dependent manner. Furthermore, we observed Tregs in the CSF from a PIIRS patient. Recent studies on PIIRS also showed Tregs in the CSF from these patients^21^. Likewise, improved survival and lower inflammatory damage in CXCR3^−/−^ and CCR2^−/−^ mice, or mice and patients treated successfully with the JAK inhibitor ruxolitinib was associated with increased Treg frequencies^21,24,26,31^, suggesting that the appropriate Treg/Teff balance helps to minimize collateral damage in the brain. Here we provide direct evidence that Tregs are critical players in the anti-fungal CNS response and that by manipulating their proportions during CNS infection, we may make a true life versus death difference (**Compare** Figs. 1d and 6d). Our data demonstrate that the predominant effect of Tregs was to restrain Teff Th1 responses (Figs. 1h–k,6c, h–j), but we also saw additional anti-inflammatory effects on other cells such as CD8^+^ T cells, microglia and moDCs. These additional effects are likely to be secondary to the predominant effect of Tregs on the CD4^+^ T cell subset, which appear very uniformly as a result any of our presented Treg manipulations or factors that Treg produce.

IL-10, a major Treg product, restrains inflammation and protects the host from brain damage during CM. Our investigation suggests the IL-10 signaling axis as a dominant mechanism by which Tregs confer neuroprotection during CM. This conclusion is supported by two lines of evidence. First, both Treg-depleted and IL-10-deficient mice exhibit markedly accelerated mortality and exacerbated neuroinflammation, underscoring the shared phenotype of uncontrolled CNS immunopathology. Second, single-cell transcriptomic profiling reveals that Tregs are the principal source of IL-10 within the infected brain. Although Tregs deploy multiple suppressive mechanisms, our data highlight IL-10 production as a critical protective factor in counteracting Th1-mediated immunopathology in this context. This finding contrasts with the prevailing view from earlier animal studies that IL-10 is detrimental in fungal infections due to suppression of fungal clearance^25, 48^. By shifting the focus to host survival and immunopathology—rather than fungal clearance—we reveal a previously underappreciated, context-specific protective function of IL-10. This insight provides a compelling explanation for the paradox observed in human CM, where elevated CSF IL-10 levels have been associated with both poor outcomes^49–51^ and improved survival^22, 52, 53^. Thus, our data suggest that the biological context of IL-10 is a crucial determinant of its effect during CM. Together, our results establish that Treg and IL-10 are central regulatory pathways in CNS immune defense and highlight their therapeutic potential as a target to mitigate lethal inflammation during fungal brain infections.

Areg on Tregs shows a complex role including modulation of Treg function on Th1 response during CM. Areg is highly expressed by Tregs, reported to enhance Treg function and synergize with IL-10 during chronic inflammation^54^. It is reported to act as a tissue repair factor^55, 56^, but its effect in inflammatory conditions in different organs is contextual^57–59^. In the brain, amphiregulin can limit pro-inflammatory responses in astrocytes in a mouse model of MS, suggesting its potential to reduce neuroinflammation and promote neuronal repair^32^. However, Areg has not been studied in infectious meningitis or cryptococcal disease, especially in the context of Treg function. Areg is highly expressed on Tregs (Fig. 5) in the brain during CM; however, Areg suppression in an Areg^flox/flox^Foxp3^Cre-ERT2^ model did not affect mouse survival or demonstrate any net effect on brain neuropathology. Unexpectedly, Areg-deletion decreased CD4^+^ T cell-IFN-γ production at the 21, 30 and 35 dpi, timepoints consistent with the presence of Tregs in the brain, and the corresponding delay in fungal clearance. This demonstrates that in CM, Areg is mostly pro-inflammatory and serves as an internal regulator of Th1-supressive Treg function. This contrasts with previous reports which demonstrated Areg enhancing Treg immunosuppressive effects^60^. Together, these data are consistent with differential functions of Areg in different organs, such as the kidney and lung where Areg is also proinflammatory^57–59^. Despite its proinflammatory effect in the context of CM, we have not observed a survival or pathological effect associated with Treg-restricted deletion of Areg. This could be explained by Areg exerting multiple biological effects, where harmful proinflammatory effects could be alleviated by other beneficial effects such as reported neuronal preservation^32^ or other yet to be explored functions of Areg.

CCR8 could be dispensable for CNS protection by Tregs in CM. Among multiple pathways known to recruit Tregs, the chemokine CCR8-CCL1 axis seems to be very important. The CCR8-CCL1 axis has been shown to induce migration and enhance the suppressive capacity of human Tregs^61–63^. In mouse EAE, CCR8^+^ Foxp3^+^ Treg subset shows increased expression of IL-10, and Granzyme B (GzmB), both linked to Treg-suppression of EAE symptoms, which mirrors the strong suppressive role of CCR8^+^ Treg in the cancer field^39^. Our data demonstrate that blocking CCR8 decreases Tregs in the brain significantly, but it cannot completely block their recruitment or block the neuroprotective function of Tregs even after CCR8 was blocked.

Low dose IL-2 immune complex therapy is a potential immunotherapy for CM. IL-2 immunotherapy has been used for autoimmune diseases, neurological diseases and cancers^64–66^. while a high dose of IL-2 administration boosts T effector cell responses^64, 65^, a specific low dose IL-2 immune complex treatment triggers more Tregs. Our data demonstrated promising improvements in inflammation, neurological deficits and survival using IL-2 immune complex treatment, including a significantly decreased CD45^+^ immune cells infiltration into the brain, significantly decreased Th1 responses while sparing Th2 and Th17 components. Clinical trials using IL-2 immunotherapy to treat cancers, autoimmune diseases and infectious diseases worldwide, support the safety and high potential for clinical application^64, 67^. Moreover, in 2008, there was a successful treatment using IL-2 therapy for persistent cryptococcal meningitis in a child with idiopathic CD4^+^ T lymphocytopenia^68^, suggesting that IL-2 immunotherapy could be safe for HIV/AIDS patients. However, in our hands IL-2 immune complex therapy also led to increased fungal burden, and thus individualized approaches would need to be made regarding the use of IL-2 and its dosing on a patient-by-patient basis. Further studies are also needed to assess how such immunotherapies could advance the treatment of other forms of infectious and autoimmune encephalitis and meningitis, to which the principles presented here could apply.

In summary, our study revealed the critical role of Tregs in the CM brain in balancing the removal of an infectious agent from the CNS while shielding the brain from inflammatory damage. In addition, we demonstrated that manipulation of Tregs can be harnessed to control the damage response as a therapeutic approach in one of the most world’s most important CNS infections. We also dissect mechanisms by which Tregs modulate immune defenses to shield the brain from damage. Our findings support the Treg pathways as a potential therapeutic target to limit brain immunopathology in life-threatening central nervous system infections.

## Materials and Methods

### Ethics Statement and human patients

All patients were seen at the National Institutes of Health Clinical Center, Bethesda, Maryland, in 2023–2024 and informed consent was obtained under an institutional review board-approved protocol (National Institute of Allergy and Infectious Diseases [NIAID] protocol 93-I-0106) for a prospective observational study examining the host genetics and immunology of cryptococcal disease in previously healthy, non-HIV infected adults. A diagnosis of CM was defined as a positive latex agglutination cryptococcal antigen or the isolation of *Cryptococcus* in one or more CSF cultures, or both.

C57BL/6 mice were purchased from The Jackson Laboratory (Bar Harbor, ME). Areg^FL/FL^ was a gift from Prof. Alexander Rudensky from Memorial Sloan Kettering Cancer Center. Foxp3^Cre-ERT2^, Areg^FL/FL^Foxp3^Cre-ERT2^ and IL10^−/−^ mice were bred and housed under specific pathogen-free conditions in the Animal Care Facility at the Veterans Affairs Ann Arbor Healthcare System. Eight-twelve-week-old mice were used for infection and were humanely euthanized by CO_2_ inhalation at the harvested time. For survival monitoring, mice were euthanized at the bodyweight loss at 20%, had persistent cranial swelling, and/or developed neurological symptoms. Tamoxifen treatment was performed as previously described with some differences^69^. Briefly, tamoxifen (Sigma, T5648) was dissolved in corn oil (Sigma, C8267) by shaking overnight at 37°C to a final concentration of 20mg/mL. Tamoxifen was administered via intraperitoneal injection at days 10, 12, 14 and 16dpi using a dose of 150mg/kg. Bacon chow was supplied during treatments. All experiments were approved by the Veterans Affairs Institutional Animal Care and Use Committee and were performed in accordance with NIH guidelines and the Guide for the Care and Use of Laboratory Animals.

### Animal infections

*C. neoformans* 52D strain, ATCC 24067 (American Type Culture Collection, Manassas, VA), was used for mouse infections in this study as our previous studies^24^. Cryptococcal strain was grown for 48 h on SDA (Sabouraud dextrose agar, SDA, Difco), and then overnight culture in SDB (SDA (Sabouraud dextrose broth, SDB, Difco). Fungal cells were washed twice in PBS, counted on a hemocytometer with trypan blue, and adjusted to a concentration of 5 × 10^6^ cells/mL for infection. Mice were anesthetized using isoflurane and then infected using 1 × 10^6^ *Cryptococcus neoformans* 52D (in 200 μL PBS) via retro-orbital intravenous injection. Serial dilutions of the *C. neoformans* suspension were plated on an SDA plate to confirm the number of viable fungi for the infection.

### Antibody blocking

To neutralize the CD4^+^CD25^+^ Tregs^70^, mice received anti-CD25 mAb (BioXcell, PC-61.5.3, #BE0012) or rat IgG1 (BioXcell, HRPN, # BE0088)) in PBS separately and repeatedly treated i.p. 200ug with anti-CD25 mAb at 0 dpi, and 100ug weekly follow-up for continuing neutralization. For CCR8 block^32^, mouse was treated using 10 μg of CCR8 mAb (Biolegend, CD198 (CCR8) Antibody, 150314) or rat IgG2b (BioLegend, RTK4530, 400602) intraperitoneally daily from 10 dpi. For IL2 complex treatment^64^, 1μg of murine IL-2 cytokine (Biolegend, 575406) was incubated with 5 μg of anti-mouse IL-2 mAb (BioXcell, JES6–1A12, #BE0043) in PBS, for 30 minutes at 37 °C prior to intraperitoneal administration to each mouse in a volume of 200μl i.p. injections of IL-2 complexes at 7, 8, 9, 11, 13, 15, 17, 19, 21, 23, 25… days post infections.

### Murine coma and behavioral scale

The murine coma and behavioral scale (MCBS) to assess the overall physical and neurological condition of infected mice was performed as previously described^71^. Briefly, mice were scored on a scale of 0 to 3 for exploration, balance, gait, body posture, coat condition, grip strength, reflexes (body, neck, pinna and footpad reflexes), and response to visual stimuli. Lower scores reflect more-pronounced symptoms.

### CFU analysis

Brains and spleens samples were harvested, homogenized, and serially diluted with distilled water. Then, 10 μL aliquots of each sample were plated on SDA in duplicates. Total colony-forming units (CFU) were calculated 48 hours incubation after plating.

### Brain leukocytes isolation

Mouse brain leukocytes isolation was performed as previously described^24^. Briefly, mice were euthanized using CO_2_ and then perfused with 10 mL PBS. The brains were aseptically removed, transferred to GentleMACs C tubes containing 5 mL of sterile digest medium (RPMI 1640 with 5% fetal bovine serum [FBS], 25 mM HEPES, GlutaMAX, penicillin-streptomycin, nonessential amino acids, sodium pyruvate, beta-mercaptoethanol, 10μL DNAse and 0.01g collagenase). The tissue was minced and then processed on a GentleMACs homogenizer (Miltenyi) to obtain the brain homogenate and filtered through a 70-μm cell strainer. Finally, microglia and brain-infiltrating leukocytes (BIL) were recovered from the 30%/70% Percoll. Isolated leukocytes were washed twice with PBS and ready to use. Total cell numbers were determined by counting live cells on a hemocytometer with trypan blue.

For cerebral spinal fluid (CSF) cells from human patients, CSF was immediately processed for flow cytometry staining as in (PMID: 40117367).

### NanoString analysis

Total RNA was isolated from the whole-brain homogenate using TRIzol (Life Technologies) and sent for NanoString nCounter analysis (NanoString Technologies)^31^. Briefly, target RNA was labeled with a capture probe and a reporter probe specific to the genes of interest (Mouse Neuropathology Panel). After hybridization, the probe-target complexes were immobilized on an imaging surface and then scanned by a fluorescence microscope. Data analysis was performed on the nSolver analysis software according to the manufacturer’s instructions and built-in statistical analyses. Pathway scores were calculated as the first principal component of the pathway genes’ normalized expression. The software will orient the scores such that increased score corresponds with increased expression in a majority of the pathway genes. Scores of the cell type characteristic genes were analyzed using the default settings from the cell type profiling algorithm in the nSolver analysis software.

### Single-cell RNA analysis

Single cell data from our previous publication was used for analysis^26^. Briefly, the single cell data were aligned to the mouse mm10 genome using the cellranger pipeline (v6.0.0). Initial single-cell expression matrices from cellranger underwent quality control: cells with <500 UMI counts, <200 detected genes, or >20% mitochondrial genes were excluded. We utilized Seurat for single-cell integration and clustering. Cell types within clusters annotated using GPTCelltype^72^.

### Gene Expression

Gene transcript levels were determined by quantitative PCR. Chemokines expression on Tregs and Teffs: Brains from mice at 21dpi were harvested and digested as mentioned above. Tregs cells were isolated using an EasySep^™^ Mouse Treg Cell Isolation Kit (StemCell Technologies, Vancouver, Canada) according to manufacturer instructions. Total RNA was isolated using the TRIzol reagent (Life Technologies), which were then converted to cDNA with Quantitech reverse-transcription kit (Qiagen) according to manufacturer instructions. Gene expression was quantified with SYBR-green amplification (Radiant Green master mix; Alkali Science) using a LightCycler 96 thermocycler (Roche). Relative gene expression was then analyzed using the 2-ΔΔCq method relative to housekeeping genes *Gapdh* and *18S* ribosomal RNA.

### Flow cytometry

Mouse brain cells were stained with fixable live/dead dye (Life Technologies), blocked with anti-CD16/32, and stained with CD45 (30-F11), CD3 (145–2C11), CD4 (GK1.5), CD8 (53–6.7), CD11b (M1/70), CD11c (N418), Ly6C (HK1.4), Ly6G (1A8), and/or major histocompatibility complex class II (MHCII, M5/114.15.2). For IFN-γ production, the cells were stimulated for 6 h with PMA (phorbol myristate acetate, PMA) and ionomycin and blocked by brefeldin A and monensin for the final 4 h. The cells were stained for extracellular markers and then fixed with fixation/permeabilization buffer (eBioscience FoxP3/Transcrition Factor staining buffer set), and intracellular staining for Foxp3 and IFN-γ. Fluorescence minus one (FMO) control was used for all experiments. Data were collected either on an LSRII cytometer (BD) or LSRFortessa (BD) and were analyzed using FlowJo (Treestar).

To assay for immune cell populations within the CSF, CSF cells and PBMCs were first blocked with Fc Receptor Binding Inhibitor (Thermo Fisher Scientific, cat. no. 14-9161-73, RRID: AB_468582) in flow staining buffer (PBS containing 1% BSA) for 15 min at 4°C and then stained with antibodies to CD45 FITC (fluorescein isothiocyanate; HI30, Thermo Fisher Scientific, cat. no. 11-0459-42, RRID: AB_10852703), CD3 PB (UCHT1, BioLegend, cat. no. 300417, RRID: AB_493094), CD4 APC-Cy7 (SK3, Biolegend), and CD25 PerCP-Cy5.5(M-A251, BD) for 30 min at 4°C. After incubation, cells were washed twice with flow staining buffer and fixed in Cytofix buffer (BD) for 30 min before washing with eBiosciences FoxP3/Transcription Factor Staining Kit (eBiosciences, Cat: 00-5521-00) 1x permeabilization buffer twice before resuspension of cell pellet in buffer containing antibody to FoxP3 PE (236A/E7, eBiosciences) overnight at 4°C. The next morning, cells were washed twice with 1x permeabilization buffer twice before resuspension of cell pellet in flow staining buffer and were immediately collected on an LSRFortessa flow cytometer (BD) with FACSDIVA software (BD) and analyzed using FlowJo software (BD). Appropriate unstained, single-color control and FMO controls were run with samples.

### Microscopy

Brain immunofluorescence stainings were performed as previous described^31^. Briefly, perfused brain tissues were isolated and fixed using 10% formalin for 48h, then 5 μm paraffin section were used for H&E and immunofluorescence staining. Primary antibodies were added at β-III tubulin antibody (1:100, Cat#: ab18207, Abcam or 1:100, Cat#: MAB1195, R&D), a synaptotagmin-7 antibody (1:100, Cat#: MA5–27654, Invitrogen), a cleaved caspase-3 (1:100, Cat#: AB3623, Sigma), and CD4 (1:100, Cat#: AB183685, Abcam) for 1 h at 37°C. The secondary antibody was added at a 1:500 dilution for 488 goat anti-Mouse (1:500, Cat# A11001, Thermofisher), 647 goat anti-Mouse (1:500, Cat# 21235, Thermofisher), 647 goat anti-Rabbit (1:500, Cat# A32733, Thermofisher), 488 goat anti-Rabbit (1:500, Cat# 11034, Thermofisher), or 555 goat anti-Rat (1:500, Cat# A21434, Thermofisher). After washing in DPBS three times (3 min per wash), ProLong Gold antifade mountant with 4ʹ,6-diamidino-2-phenylindole (DAPI; Life Technologies) was used for mounting the coverslips. The autofluorescence was quenched by Vector’s true view kit (Vector Laboratories) as per the manufacturer’s protocol before mounting with mounting medium. Samples were visualized using a KEYENCE microscope BZ-X800 series. Images were captured by the camera provided by the vendor by using a ×20 objective 0.45 numerical aperture (NA) Nikon. Images were deconvolved for enhancing the clarity of the images. Images were analyzed by Fiji, and the numbers of CD4^+^ T cells, and MFIs of synaptotagmin-7 (Syt7) and cleaved caspase-3 were calculated around the area infected by *Cryptococcus*. Areas that are 500 square microns around the cryptococcal lesions were analyzed. All the images were captured under the same settings.

### Statistical analysis

Statistical analysis was performed using GraphPad Prism v10 software with Student’s t-test, or log-rank test or analysis of variance (ANOVA) plus Dunnett’s multiple comparison test for multiple comparisons. *, P < 0.05; **, P < 0.01; ***, P < 0.005; ****, P < 0.001.

## Extended Data

## Supplementary Material

Supplement 1 Extended data table NanoString and Single cell analyses.
^a^ Protein-protein interactions for NanoString analysis.
^b^ Top 10 markers for T cell clusters.
^c^ Differentially expressed genes between Tregs and T effector cells.

Supplementary Files

This is a list of supplementary files associated with this preprint. Click to download.

- ExtendeddatatableNanoStringandSinglecellanalyses.xlsx
